# Optimization of recombinant bacteria expressing dsRNA to enhance insecticidal activity against a lepidopteran insect, *Spodoptera exigua*

**DOI:** 10.1371/journal.pone.0183054

**Published:** 2017-08-11

**Authors:** Mohammad Vatanparast, Yonggyun Kim

**Affiliations:** 1 Department of Plant Protection, College of Agriculture, University of Bu-Ali Sina, Hamedan, Iran; 2 Department of Plant Medicals, Andong National University, Andong, Korea; Institute of Plant Physiology and Ecology Shanghai Institutes for Biological Sciences, CHINA

## Abstract

Double-stranded RNA (dsRNA) has been applied to control insect pests due to its induction of RNA interference (RNAi) of a specific target gene expression. However, developing dsRNA-based insecticidal agent has been a great challenge especially against lepidopteran insect pests due to variations in RNAi efficiency. The objective of this study was to screen genes of chymotrypsins (SeCHYs) essential for the survival of the beet armyworm, *Spodoptera exigua*, to construct insecticidal dsRNA. In addition, an optimal oral delivery method was developed using recombinant bacteria. At least 7 SeCHY genes were predicted from *S*. *exigua* transcriptomes. Subsequent analyses indicated that *SeCHY2* was widely expressed in different developmental stages and larval tissues by RT-PCR and its expression knockdown by RNAi caused high mortality along with immunosuppression. However, a large amount of dsRNA was required to efficiently kill late instars of *S*. *exigua* because of high RNase activity in their midgut lumen. To minimize dsRNA degradation, bacterial expression and formulation of dsRNA were performed in HT115 *Escherichia coli* using L4440 expression vector. dsRNA (300 bp) specific to *SeCHY2* overexpressed in *E*. *coli* was toxic to *S*. *exigua* larvae after oral administration. To enhance dsRNA release from *E*. *coli*, bacterial cells were sonicated before oral administration. RNAi efficiency of sonicated bacteria was significantly increased, causing higher larval mortality at oral administration. Moreover, targeting young larvae possessing weak RNase activity in the midgut lumen significantly enhanced RNAi efficiency and subsequent insecticidal activity against *S*. *exigua*.

## 1. Introduction

RNA interference (RNAi) regulates gene expression at post-transcriptional level by reducing the half-life of specific mRNA or interfering with translational efficiency [1]. RNAi technology has been widely used in functional genomics through specific knockdown of target gene expression [2]. RNAi using double-stranded RNA (dsRNA) has been proposed to be an alternative way to develop novel insecticidal agents [3]. Indeed, two pioneering research studies using transgenic crops expressing dsRNA support dsRNA-based insect pest control strategy [4,5]. However, developing efficient dsRNA insecticidal agents against lepidopteran pests is challenging due to variation in RNAi efficiency [6]. The beet armyworm, *Spodoptera exigua*, is a major pest of vegetable crops. Insecticide resistance development against many commercial chemical insecticides makes it difficult to control the insect pest [7–9]. Therefore, alternative control method is needed. To this end, dsRNA strategy has been considered for controlling *S*. *exigua*. At least three different genes have been tested as RNAi targets by different research groups. Chitin synthase, a metabolic gene, has been successfully knock-downed by its specific dsRNA, causing significant mortality of *S*. *exigua* [10]. Ecdysone receptor, a developmental gene, has also been tested as RNAi target through dsRNA strategy, causing significant mortality [11]. To interfere with cell-cell interaction, a β-subunit of integrin has also been knocked-down by dsRNA, resulting in significant mortality of *S*. *exigua* [2]. These results support that it is feasible to use dsRNA to control *S*. *exigua*. However, to improve insecticidal efficiency, better target gene and efficient dsRNA delivery method are needed.

Digestive proteases may be excellent target genes for dsRNA to control *S*. *exigua* because plants use protease inhibitors to protect them against insect herbivores [12]. Digestive proteases in lepidopteran insects include serine proteases, cysteine proteases, carboxypeptidases, and aminopeptidases, in which serine proteases play predominant (~95%) roles in digestion of diet proteins [13]. Trypsin and chymotrypsin are serine proteases identified in midgut transcriptomes of several lepidopteran insects [14]. For example, there are 120 serine proteases in the genome of diamondback moth, *Plutella xylostella*, including 38 trypsins and 8 chymotrypsins [15]. Transcriptome analysis of the Asian corn borer, *Ostrinia furnacalis*, has identified 19 trypsins and 7 chymotrypsins [16]. RNAi of these serine proteases has resulted in significant mortalities along with reduced body size, suggesting that the malnutritional effect is probably due to the lack of digestive enzymes available [16]. These results suggest that midgut digestive serine proteases might be good targets for constructing insecticidal dsRNA to enhance insecticidal efficacy against *S*. *exigua*.

To maximize RNAi efficiency and subsequent insecticidal activity of dsRNA, an optimal serine protease gene was selected and applied to construct an efficient expression and delivery system. First, this study showed that chymotrypsins (SeCHYs) were crucial for survival and development of *S*. *exigua* larvae. SeCHYs were then subjected to screening as RNAi targets based on their expression levels and RNAi efficacies. Second, limiting factor of dsRNA was determined through oral administration. Third, to prevent dsRNA degradation and supply large amounts of dsRNA, a recombinant bacterial expression system was used to produce dsRNA. Fourth, bacterial delivery system was modified to facilitate dsRNA release from recombinant bacteria. Finally, the optimal developmental stage of *S*. *exigua* for effective control by dsRNA was determined.

## 2. Materials and methods

### 2.1. Insect rearing

Beet armyworm larvae were reared with an artificial diet [17] at controlled condition (25°C, 16:8 h L:D photoperiod, and 60 ± 5% relative humidity). Adults were supplied with 10% sucrose solution. Larval instars (L1-L5) were determined based on head capsule sizes [17]. Different larval tissues were isolated from 3 days old L5 instars.

### 2.2. Entomopathogenic bacterial culture

Two entomopathogenic bacteria were used in this study. *Xenorhabdus hominickii* ANU101 [18] was cultured in Luria-Bertani (LB) medium (10 g Bacto tryptone, 5 g Bacto yeast extract, and 10 g NaCl in 1 L H_2_O) for 48 h at 28°C with shaking (225 rpm). To kill *X*. *hominickii*, high temperature of 95°C for 20 min was used. *Bacillus thuringiensis* ssp. *aizawai* (Bt, an isolate of commercial product of Xentari^®^) was cultured in LB medium at 28°C for 5 days with shaking (225 rpm). It was then kept at 4°C for 2 days to allow spore formation [19]. Resulting bacteria were counted with a hemocytometer (Neubauer, Marienfeld, Germany) at 200 x magnification under a phase contrast microscope (BX41, Olympus, Tokyo, Japan). Bacterial concentrations were expressed as cells (for *X*. *hominickii*) or spores (for Bt) per mL.

### 2.3. Bioassay to determine the effect of protease inhibitors on survival of *S*. *exigua* larvae

Five different treatments (four individual inhibitors and their mixture) were used to assess their effect on the survival of *S*. *exigua* larvae: (1) chymostatin specific to α-, β-, γ-, δ-CHY, papain, cathepsin- A, B, and D; (2) tosyl phenylalanyl chloromethyl ketone (TPCK) specific to CHY, cerastocytin, papain, ficin, but not trypsin; (3) tosyl-L-lysyl-chloromethane hydrochloride (TLCK) specific to trypsin, cerastocytin, but not CHY; (4) cathepsin III inhibitor (CATH) specific to cathepsin, and (5) an inhibitor mixture with equal mass ratio of four inhibitors. All inhibitors were dissolved in dimethyl sulfoxide (DMSO) to prepare stock solutions at 50, 500, and 5,000 ppm. L3 larvae were fed diets soaked in different inhibitors for 5 days. Treated larvae were then supplied with untreated diet for 3 days. Survival rates were measured at 8 days after the initiation of treatment. Each treatment was replicated three times. For each replication, 10 larvae were used. As control, diet was soaked in 10% DMSO without any inhibitor.

### 2.4. Bioinformatics

A CHY-like gene was identified from midgut transcriptome [20]. Using its gene sequence (GenBank accession number: AY820894.1) as query, BLAST search was performed against SPODOBASE database. Blasted sequences (SeCHYs) were re-annotated using Blast P in NCBI GenBank database. Predicted amino acid sequences were then aligned using Clustal W (DNASTAR Version 7.0). Phylogenetic trees were constructed with Neighbor-joining method and Poisson correction model (1,000 bootstrap repetitions to check for repeatability of results) using MEGA 6.06 software (www.megasoftware.net).

### 2.5. RNA extraction, RT-PCR, and qPCR

RNA was extracted from whole body of *S*. *exigua* at different developmental stages (100 eggs, 20 young larvae (L1-L3), three L4 larvae, one L5 larva, one pupa, and one adult for each extraction). Total RNA was extracted using Trizol reagent (Invitrogen, Carlsbad, CA, USA) according to the manufacturer’s instruction. The extracted RNA was treated with RNase-free DNase (Bioneer, Seoul, Korea) to degrade any genomic DNA contamination. RNA extract (1 μg per reaction) was used for cDNA synthesis using RT-premix (Intron Biotechnology, Seoul, Korea). The synthesized cDNA was used as template for PCR amplification with SeCHYs-specific forward and reverse primers (S1 Table). Expression of a ribosomal gene, *RL32*, was assessed with its gene-specific primers (S1 Table) to determine cDNA integrity. After an initial denaturation at 95°C for 1 min, PCR was performed with 35 cycles of denaturation at 95°C for 1 min, annealing at 50°C for 1 min, and extension at 72°C for 1 min. PCR reaction was terminated with a final extension step at 72°C for 10 min.

Quantitative PCR (qPCR) was performed using SYBR Green Real time PCR master mixture (Toyobo, Osaka, Japan) on a CFX Connect Real-Time PCR System according to the manufacture’s instruction. The reaction mixture (20 μL) included 10 pmol of primers used in RT-PCR as described above and 100 ng of cDNA template. After activating Hot-start Taq DNA polymerase at 95°C for 1 min, the reaction was amplified with 39 cycles of denaturation at 95°C for 1 min, annealing at 50°C for 1 min, and extension at 72°C for 1 min. It was then finished with a final extension step at 72°C for 10 min. Fluorescence values were measured and amplification plots were generated in real time by CFX Manager. *RL32* was used as control for qPCR. Each cycle was scanned to quantify PCR products. Melting curves of PCR products were analyzed to confirm single products. Each treatment was replicated with three independent biological sample preparations. Quantitative analysis of gene expression was performed using comparative CT method [21].

### 2.6. Silencing *SeCHY* expression by RNA interference (RNAi)

RNAi was performed using dsRNA prepared with Megascript RNAi Kit (Ambion, Austin, TX, USA) according to the manufacturer’s instruction. Briefly, partial SeCHY genes of about 300 bp were amplified with primers including T7 promoter sequence at the 5ʹ end of gene-specific primers (ʻdsSeCHYsʼ, S1 Table). These dsRNAs were synthesized at 37°C for 4 h and then left at 70°C for 5 min to inactivate T7 RNA polymerase.

For dsRNA injection, 3 µL of dsSeCHY2 was injected into hemocoel of each L3, L4, or L5 instar larva with a micro-syringe (Hamilton, Reno, Nevada, USA). For dsRNA oral delivery, artificial diet cubes (~ 1 cm^3^) covered with a predetermined amount of dsRNA were used to feed larvae. After complete consumption, untreated diet was supplied. Concentration of dsRNA used for treatment was calculated by dividing treated dsRNA dose by the number of test larvae per diet. As dsRNA control (dsCON) for both injection and feeding bioassays, a 520 bp fragment of enhanced green fluorescence protein (EGFP) was used to synthesize dsCON. For each treatment, 10 larvae were used. Each treatment was replicated three times.

### 2.7. Enzyme activity assay of SeCHY

Specific protease enzyme activity of SeCHY was determined using a synthetic peptide substrate SAAPFpNA (N-succinyl-alanine-alanine-proline-phenylalanine-*p*-nitroanilide) according to published method [22]. Briefly, 5 μL of 1 mM substrate was added to 10 μL enzyme extract. Then 85 μL of 50 mM Tris-HCl buffer (pH 8.0) was added. After 30 min of incubation at room temperature, its absorbance was measured at wavelength of 405 nm.

Detection of CHY enzyme activity in polyacrylamide gel electrophoresis (PAGE) was performed using an overlay technique following the method of Vinokorov et al. [23]. Briefly, enzyme extract (15 μL) was diluted in electrophoresis sample buffer (62.5 mM Tris-HCl, pH 6.8, 10% [v/v] glycerol, 0.01% [w/v] bromophenol blue) and loaded into wells. After native PAGE using 10% gel at 85 V for 2.5 h, the gel was soaked in 50 mM Tris-HCl buffer (pH 8.0) for 10 min. After removing the buffer, the gel was covered with a nitrocellulose membrane (0.45 μm pore size) presoaked in substrate solution (SAAPFpNA 1 mg mL^− 1^) for 40 min and slightly air-dried. The gel and membrane were incubated at 37°C until faint yellow bands became visible on the membrane. The membrane was then removed and liberated pNA was diazotized by subsequent incubation (5 min each) in 0.1% sodium nitrite, 0.5% ammonium sulfamate, and 0.05% N-(1-naphthyl) ethylenediamine. After pink bands denoting CHY enzyme activity appeared, membranes were scanned with BIORAD device (Molecular imager Gel DOC^TM^ XR+ imaging system). SAAPFpNA and N-(1-naphthyl) ethylenediamine were purchased from Sigma-Aldrich Korea (Seoul, Korea).

### 2.8. Hemocyte nodulation assay and bacterial pathogenicity test

dsSeCHY2 was injected into each L3 larva at dose of 3, 6, or 10 μg. Larvae were then reared with diet at 25°C for 48 h. At 48 h after dsRNA treatment, 5 × 10^5^ cells of heat-killed *X*. *hominickii* was injected into each larva and incubated at 25°C for 8 h. After dissection under a stereomicroscope (Stemi SV11, Ziess, Jena, Germany), the number of nodules was counted at 60 x magnification. Nodules at gut, trachea, and fat body were observed. Each treatment was replicated with 10 larvae.

For bacterial pathogenicity test, Bt was used. At 48 h after injection of dsSeCHY2, treated L5 larvae were fed with 10^8^ spores/mL of Bt. For Bt treatment, diet-dipping method [2] was used. Each treatment was replicated three times. For each replication, 10 larvae were used.

### 2.9. *In vitro* dsRNA degradation assay

Hemolymph was collected by cutting abdominal prolog of L5 larvae followed by centrifugation at 1,000 × *g* for 5 min. The supernatant plasma was used for dsRNA degradation assay. For extraction of midgut juice, after chilling L1-L5 larvae on ice for 5 min, their midguts were dissected. Gut content was extracted by squeezing followed by centrifugation at 15,000 × *g* for 15 min. The resulting supernatant was used for dsRNA degradation assay.

For dsRNA degradation assay, 10 μL of tissue extract was mixed with 5 μL of dsSeCHY2 (500 ng/μL) and incubated at 25°C for different time periods. All reaction mixtures were then subjected to 1% agarose gel electrophoresis using 1X TAE buffer (40 mM Tris-acetate, 1 mM EDTA, pH 8.0). dsRNA was then visualized with EcoDye^TM^ DNA staining solution (SolGent, Daejeon, Korea). For each reaction, 1 μL (4 U/μL) of RNase A inhibitor (QIAGEN, Hilden, Germany) was used.

### 2.10. Cloning and overexpression of dsRNA specific to SeCHY2 in bacterial expression system

L4440 plasmid [24] comprising two T7 promoters in an inverted orientation flanking multiple cloning sites was used to clone partial SeCHY2 (‘dsSeCHY2’). Restriction sites of *Spe*I and *Hind*III were chosen to clone dsSeCHY because these sites were not present in SeCHY2 via GeneQuest (DNASTAR). The target fragment was ligated to L4440 plasmid using T4 DNA ligase (Promega, Madison, WI, USA). The recombinant vector, L4440-dsSeCHY2, was then transformed to *Escherichia coli* HT115 (DE3) lacking RNase III by electroporation. Single bacterial colonies were cultured in LB at 37°C with shaking (at 225 rpm) for 15 h. Cultured broth (5 mL) was then added to 500 mL of fresh LB medium containing 100 μg/mL ampicillin and cultured at 37°C until late exponential bacterial growth phase with OD_600_ = 0.6–0.7. Expression of T7 RNA polymerase was induced by adding 0.4 mM (final concentration) of isopropyl-β-D-1-thiogalactopyranoside (IPTG). Bacteria were then incubated at 37°C for an additional 4 h. Total bacterial RNA was extracted with Trizol reagent and the presence of the synthesized dsRNA was confirmed by electrophoresis using 1% agarose gel with 1 x TAE buffer.

For bioassay to assess insecticidal activity of recombinant bacteria, cultured broth overexpressing dsSeCHY2 was centrifuged at 7,000 × *g* for 10 min and the resulting cell pellet was re-suspended in distilled water.

Quantification of dsRNA amounts of recombinant bacteria followed the method described by Kim et al. [2]. Briefly, a standard curve of dsRNA quantification was generated using known amounts of dsSeCHY2 synthesized by *in vitro* transcription method with MEGAscript RNAi kit. Gel band intensities were quantified in pixels with an image analyzer (Image Lab™ Software, Bio-Rad Korea, Seoul, Korea).

### 2.11. Pretreatment of recombinant bacteria expressing dsSeCHY2

After over-expression and re-suspending bacterial cells in distilled water, two different pretreatments were used to facilitate the release of dsSeCHY2 from transformed bacteria. One was heat treatment at 95°C for 10 min. The other was sonication treatment using an ultrasonicator (Bandelin Sonoplus, Berlin, Germany) at 95% intensity with 10 min burst. The resulting bacterial viability was checked by plating 100 μL of treated bacterial sample onto LB plate containing ampicillin (100 μg/mL).

### 2.12. Feeding bioassays with recombinant bacteria expressing dsSeCHY2

For bioassay to determine insecticidal activity of recombinant bacteria, L4 larvae of *S*. *exigua* were used in a diet-dipping feeding assay. A piece of artificial diet (1 cm × 1 cm × 2 mm) was covered with 10^7^ bacterial cells in 100 μL. As control, non-recombinant HT115 bacteria were used for the feeding assay. After complete consumption (less than 24 h) of treated diet, larvae were fed with normal fresh diet for growth. Each treatment was replicated three times. For each replication, 10 larvae were used.

### 2.13. Data analysis

All studies were performed in three independent biological replicates. Data were plotted using Sigma plot 10.0 (Systat Software, Germany). Means were compared by Student’s *t* test and least squared difference (LSD) test of one way analysis of variance (ANOVA) using PROC GLM of SAS program. Significance was set at Type I error = 0.05.

## 3. Results

### 3.1. High susceptibility of *S*. *exigua* larvae to CHY inhibitor

**Fig 1:**
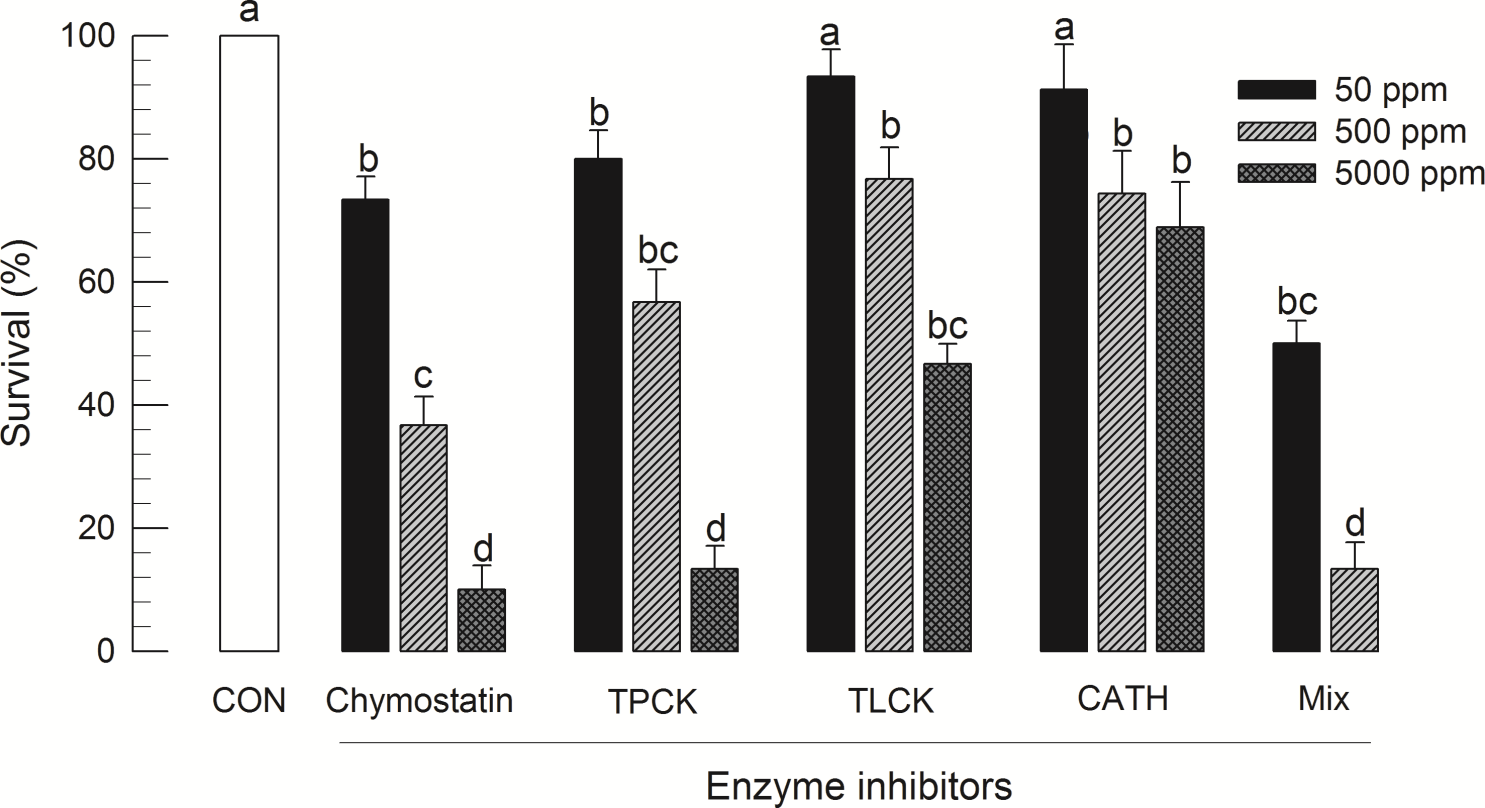
Influence of different digestive enzyme inhibitors on survival of *S*. *exigua* larvae. L3 larvae were treated with diet soaked in different concentrations of inhibitors for 5 days. Treated larvae were then reared with untreated diet for 3 days. Survival rates were determined at 8 days after treatment. Each treatment was replicated three times. For each replication, 10 larvae were used. ʻCONʼ represents solvent treatment without inhibitor. Inhibitors include chymostatin specific to α-, β-, γ-, δ- chymotrypsin, papain, cathepsin A, B and D; TPCK (tosyl phenylalanyl chloromethyl ketone) specific to chymotrypsin, cerastocytin, papain, ficin, but not trypsin; TLCK (tosyl-L-lysyl-chloromethane hydrochloride) specific to trypsin, cerastocytin, but not chymotrypsin; cathepsin III inhibitor (CATH) specific to cathepsin, and inhibitor mixture of the four inhibitors at equal mass. Different letters above standard deviation bars indicate significant difference among means at Type I error = 0.05 (LSD test).

Different digestive enzyme inhibitors were orally fed to larvae of *S*. *exigua* (Fig 1). Four different inhibitors (chymostatin or TPCK for chymotrypsin, TLCK for trypsin, and cathepsin inhibitor III (ʻCATHʼ) for cathepsin) known to specifically inhibit different digestion enzymes were used. All four inhibitors or their mixture caused significant mortalities of *S*. *exigua* larvae. However, different results were observed for various inhibitors. Chymotrypsin inhibitors (chymostatin and TPCK) were more potent than TLCK or CATH. This suggests that chymotrypsin enzyme activity might be critically required for the survival and growth of *S*. *exigua*.

### 3.2. Screening of SeCHY genes based on RNAi efficiency

A total of 23 putative SeCHY genes were collected from Spodobase (a genome database of *Spodoptera* species) (Table 1). All these genes were re-annotated with current (April 19, 2017) GenBank database using Blast P to confirm their identities. All of them matched with CHY or serine proteases significantly (< E-20). Among these candidates, genes containing full open reading frame (ORF) were selected and aligned to construct a phylogenetic tree (S1 Fig). Results of phylogenetic analysis revealed 4 subgroups. From each subgroup, one or two genes were selected. Subsequently, seven different CHY genes (SeCHY2, SeCHY3, SeCHY4, SeCHY6, SeCHY10, SeCHY18, and SeCHY19) were used in this study.

**Table 1:** Putative chymotrypsin (CHY) genes of *S*. *exigua* obtained from Spodobase (http://bioweb.ensam.inra.fr/spodobase/), open reading frame (ORF) sizes, and annotation to GenBank using Blast P.

Gene	ID	DNA (bp)	Protein (AA)*	Annotation (species)	E value
SeCHY1	Se1E46224	336	-	Midgut CHY (*Mamestra configurata)*	8e-22
SeCHY2	Se1E21589	935	279	Serine protease 33 (*M*. *configurata*)	e-176
SeCHY3	Se1E09736	976	285	Serine protease 33 (*M*. *configurata*)	e-102
SeCHY4	Se1E08718	837	263	Serine protease 33 (*M*. *configurata*)	2e-98
SeCHY5	Se1E21499	973	190	Serine protease 33 (*M*. *configurata*)	e-174
SeCHY6	Se1E18155	973	280	Serine protease 33 (*M*. *configurata*)	e-123
SeCHY7	Se1E26373	519	149	Midgut CHY (*S*. *exigua)*	2e-27
SeCHY8	Se1E10714	964	-	Serine protease 33 (*M*. *configurata*)	2e-86
SeCHY9	Se1E10614	964	212	Serine protease 33 (*M*. *configurata*)	1e-72
SeCHY10	Se1E19734	1119	280	Serine protease 33 (*M*. *configurata*)	e-111
SeCHY11	Se1E09294	606	-	Serine protease 33 (*M*. *configurata*)	9e-30
SeCHY12	Se1E13545	427	-	Serine protease 33 (*M*. *configurata*)	7e-54
SeCHY13	Se1E23101	589	-	Serine protease 33 (*M*. *configurata*)	1e-50
SeCHY14	Se1E46894	404	-	CHY-like protease C1 (*Heliothis virescens*)	5e-49
SeCHY15	Se1E55261	404	-	CHY-like protease C1 (*H*. *virescens*)	2e-22
SeCHY16	Se1E25582	450	142	Serine protease 33 (*M*. *configurata*)	1e-29
SeCHY17	Se1E38960	403	-	Serine protease 33 (*M*. *configurata*)	1e-29
SeCHY18	Se1E18416	992	295	CHY-like protein 2 (*S*. *litura*)	e-129
SeCHY19	Se1E18618	907	267	CHY-like serine protease 14 (*Ostrinia nubilalis*)	4e-98
SeCHY20	Se1E13930	889	-	CHY-like serine protease 14 (*O*. *nubilalis*)	2e-36
SeCHY21	Se1E47389	290	-	Serine protease 120 (*Danaus plexippus*)	4e-19
SeCHY22	Se1E38868	487	-	Serine protease 33 (*M*. *configurata*)	2e-50
SeCHY23	Se1E13543	972	296	Serine protease 36 (*M*. *configurata*)	e-108

*Hyphens indicate partial ORF.

**Fig 2:**
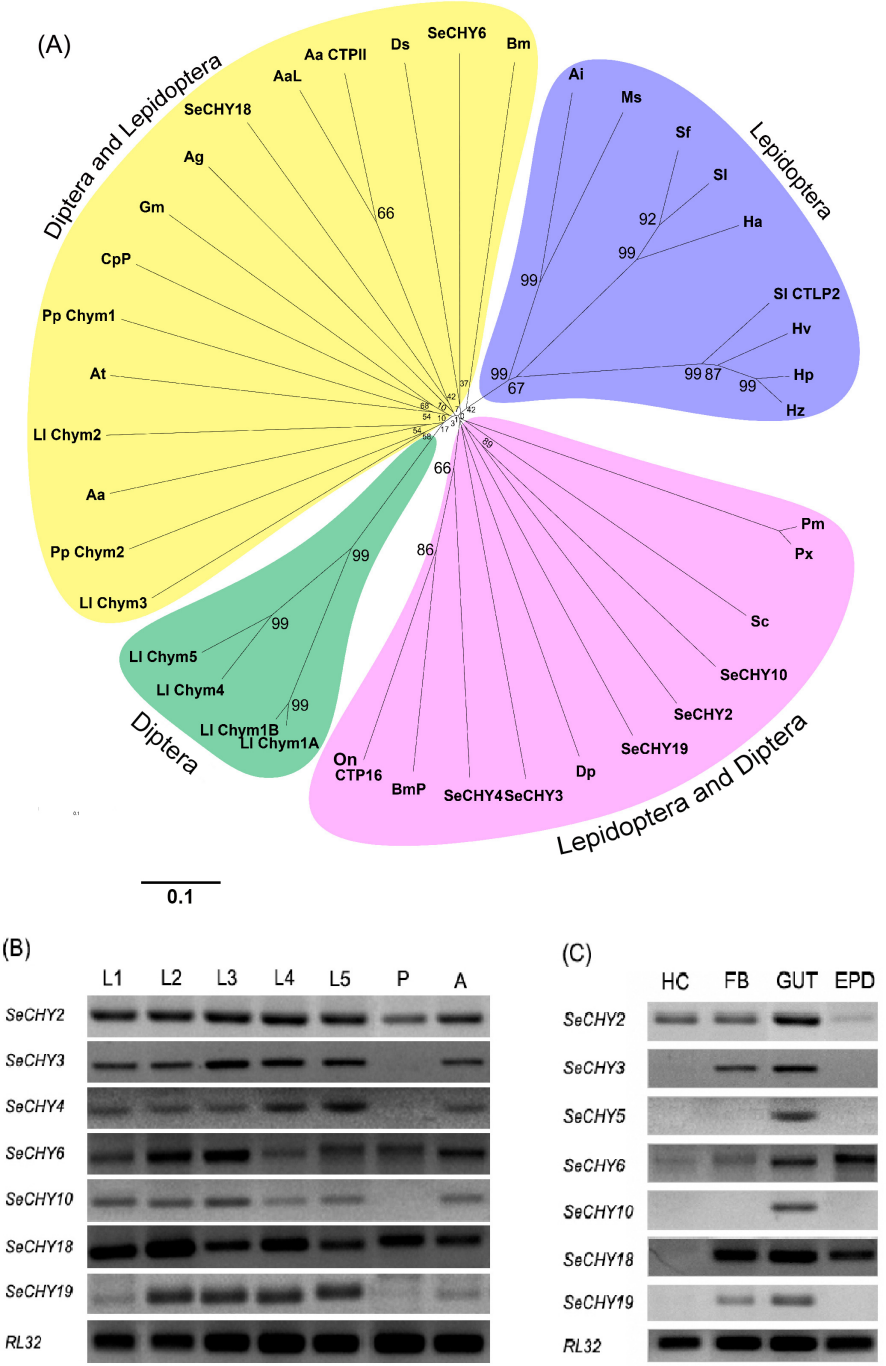
Expression analysis of 7 chymotrypsin (CHY) genes selected from *S*. *exigua*. (A) Phylogenetic tree analysis with other insect CHYs. Sequence alignment was performed with Clustal W program. The tree was constructed with MEGA 6.0. Each node contains bootstrap value after 1,000 replications. GenBank accession numbers of CHY genes other than *S*. *exigua* CHY (SeCHY) genes are denoted in S2 Table. RT-PCR analysis of SeCHYs in different developmental stages (B) and tissues (C). RL32 was used to validate cDNA integrity. Different developmental stages included larval instars (ʻL1-L5ʼ), pupa (ʻPʼ), and adult (ʻAʼ). Different tissues included hemocyte (ʻHCʼ), fat body (ʻFBʼ), midgut (ʻGUTʼ), and epidermis (ʻEPDʼ).

These seven SeCHY genes were then aligned and phylogenetically analyzed with other known insect CHY genes (Fig 2A). SeCHY6 and SeCHY18 were separated from the other five SeCHY genes. All seven SeCHY genes were expressed in *S*. *exigua*. However, their expression patterns were different (Fig 2B). Among these seven genes, only SeCHY2 was expressed in all developmental stages tested. SeCHY2 was also differentially expressed in various larval tissues. It was highly expressed in hemocyte, fat body, and midgut, but weakly expressed in epidermis (Fig 2C).

**Fig 3:**
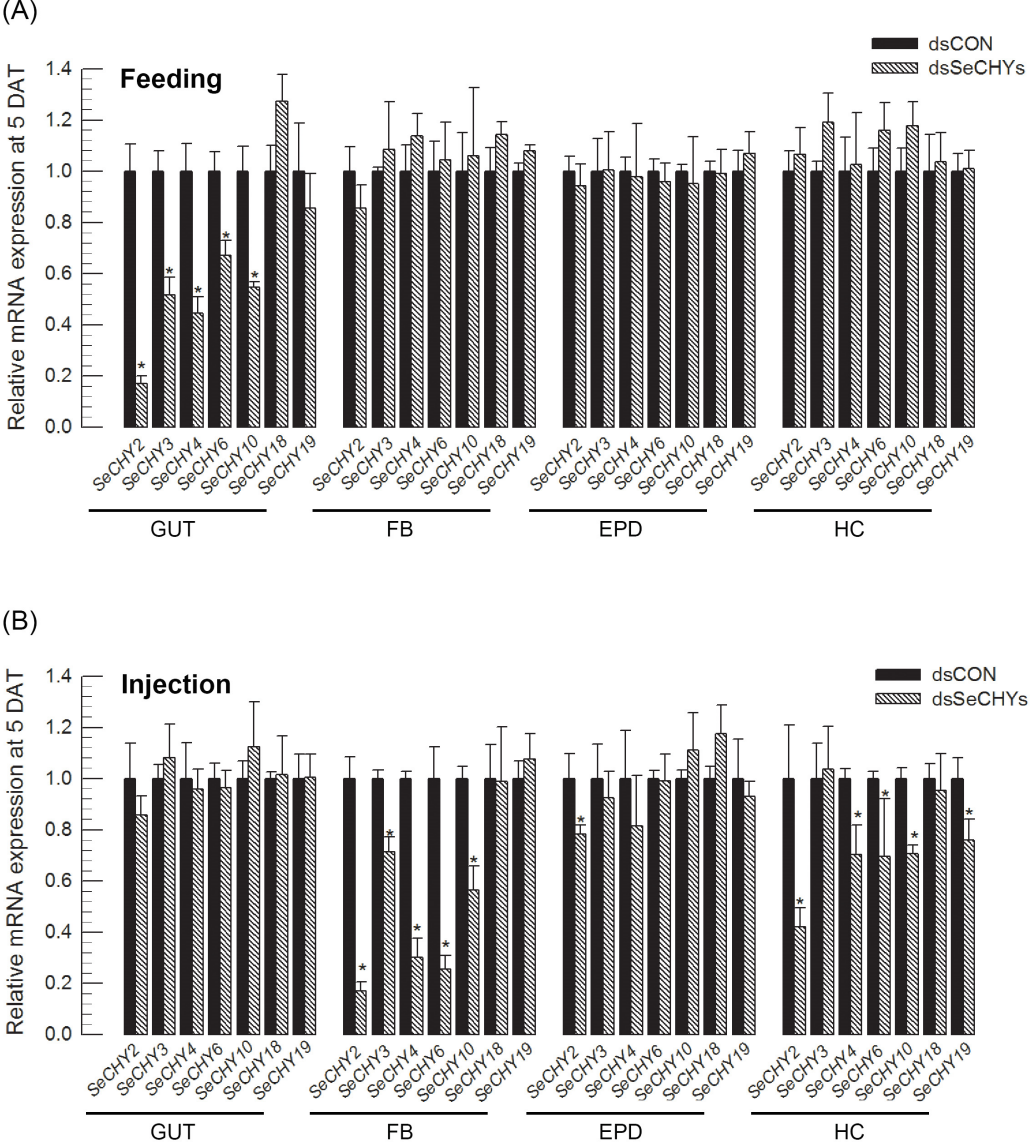
Screening of 7 different chymotrypsin (SeCHY) genes of *S*. *exigua* with respect to RNA interference (RNAi) efficiency. All dsRNAs were constructed at ~ 300 bp and injected to each L4 larva at 6 μg. Each treatment was replicated with three different insects. Control dsRNA (ʻdsCONʼ) used EGFP gene. At 48 h post injection, each SeCHY mRNA level was measured by RT-qPCR. mRNA levels were normalized by *RL32* gene expression. Relative mRNA levels were calculated based on mRNA level of dsCON treatment in each SeCHY. Asterisk (*) indicates significant difference compared to control level at Type I error = 0.05 (Student’s t test).

RNAi efficiencies of specific dsRNAs targeting the seven SeCHY genes were compared (Fig 3). These dsRNAs were applied by either feeding or injection. In feeding assay, only gut tissues exhibited significant RNAi efficacies for some SeCHY genes (Fig 3A). In injection assay, specific RNAi efficacies were observed in fat body, epidermis, and hemocyte, but not in midgut (Fig 3B). In both feeding and injection assays, SeCHY2 was the most effective in suppressing target mRNA levels. Results of these screening experiments indicated that SeCHY2 gene might be optimal to construct lethal dsRNA for *S*. *exigua*.

### 3.3. dsRNA specific to SeCHY2 possesses potent insecticidal activity against *S*. *exigua* through oral delivery

**Fig 4:**
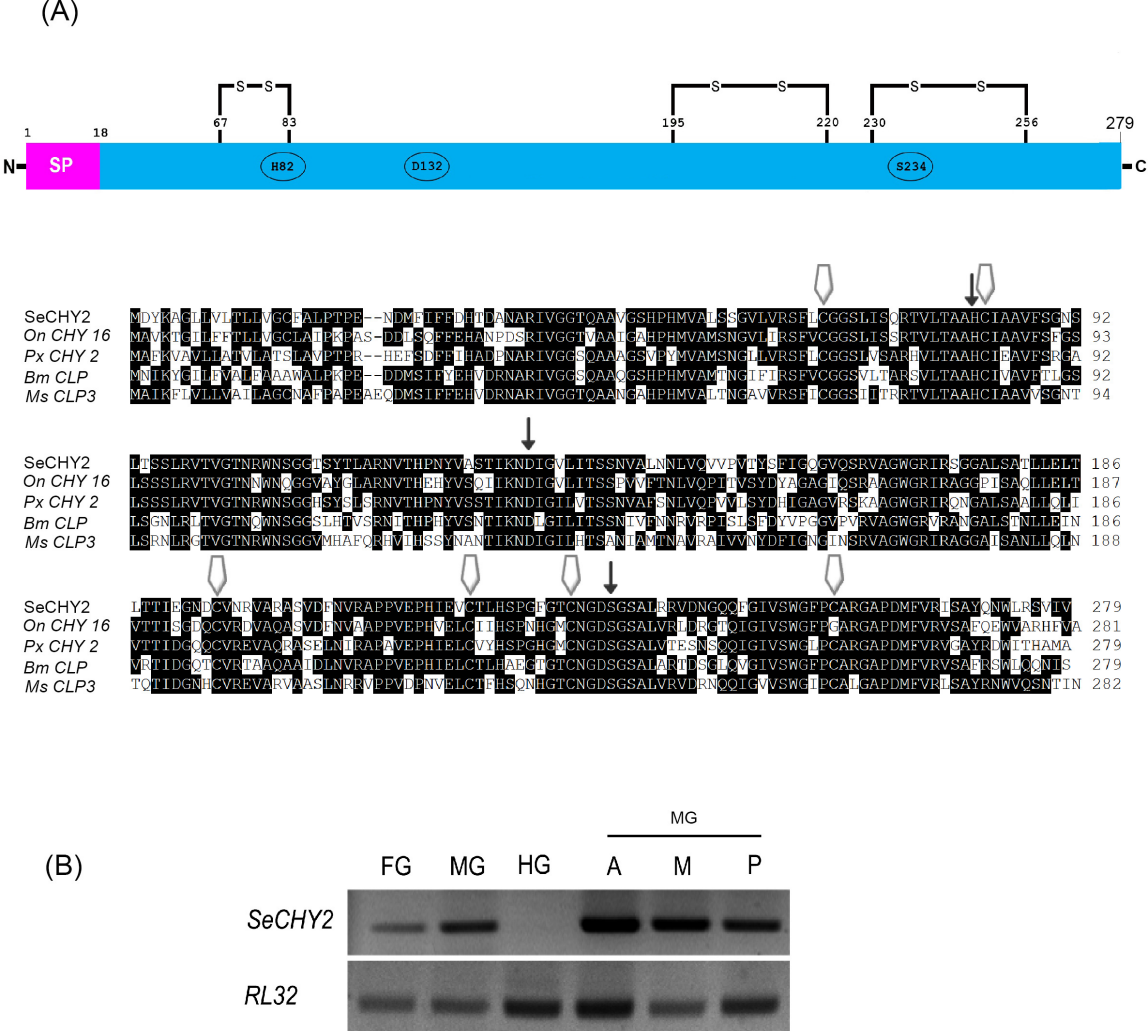
Protein structure and expression profile of SeCHY2. (A) Prediction of signature motifs of chymotrypsin. Three thin arrows indicate catalytic triad. Six thick arrows indicate conserved cysteine residues. Asterisk indicates the cleavage site of signal peptide. For sequence alignment, Clustal W program of MegAlign (DNASTAR, Version 7.0) was used. GenBank accession numbers of well-known chymotrypsin amino acid are denoted in S1 Table. (B) Expression in digestive tract: foregut (ʻFGʼ), midgut (ʻMGʼ), and hindgut (ʻHGʼ). MG was further separated into three parts: anterior (ʻAʼ), middle (ʻMʼ), and posterior (ʻPʼ). *RL32* was used to validate cDNA integrity.

Molecular structure of SeCHY was further analyzed (Fig 4). SeCHY2 shared high sequence homologies with other lepidopteran CHYs. Sequence alignment of lepidopteran CHY genes showed the following highly conserved motifs: 6 cysteine residues presumably for disulfide bond formation and 3 catalytic residues (His, Asp, and Ser) (Fig 4A). In addition, SeCHY2 was predicted to have a signal peptide that could be released from its expressing cells. Expression of SeCHY2 in all digestive tract was further analyzed. Results showed that it was expressed in foregut and midgut, but not in hindgut (Fig 4B). In the midgut, SeCHY2 was expressed in all areas.

**Fig 5:**
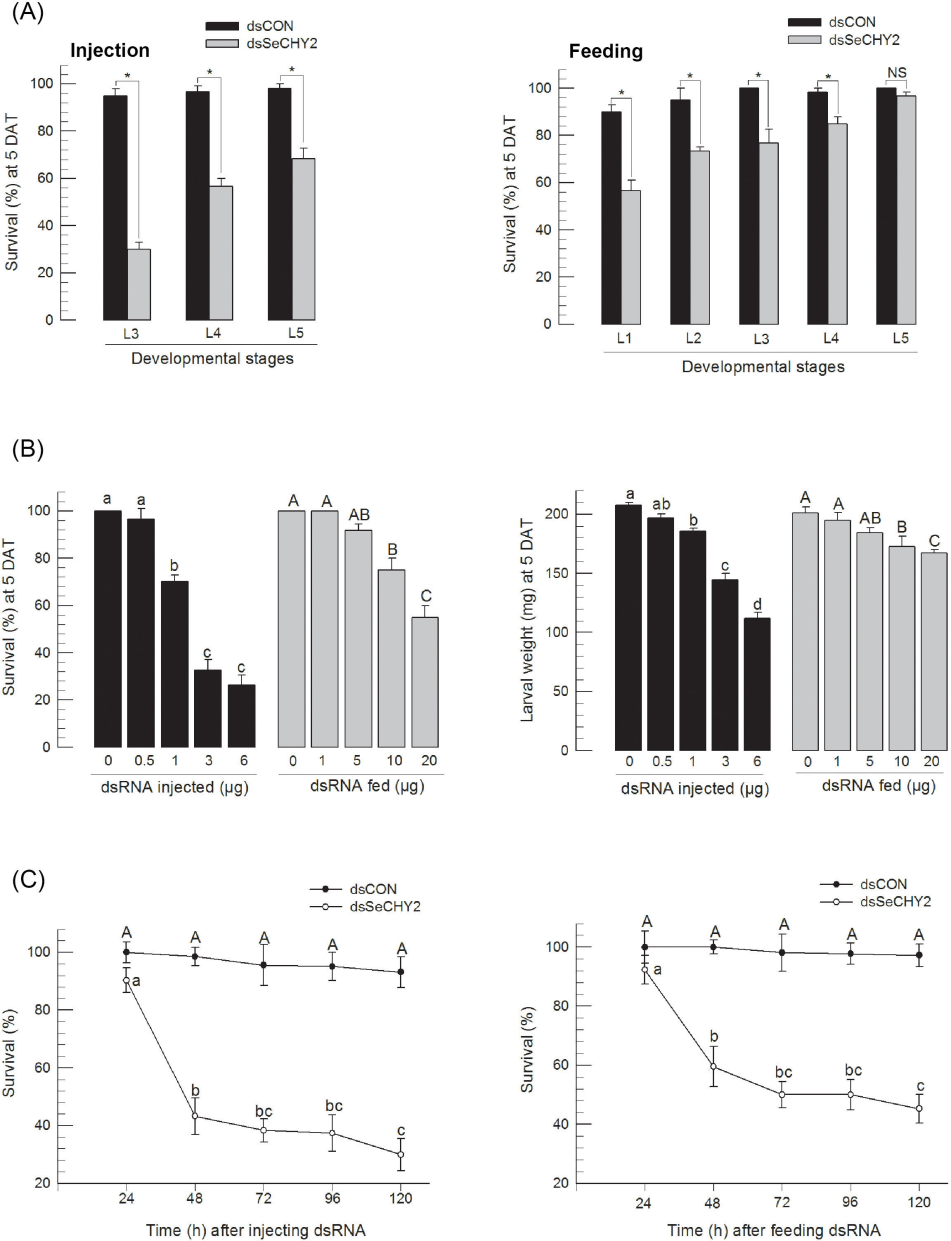
Insecticidal activity of dsRNA specific to SeCHY2 (dsSeCHY2) against different larval instars of *S*. *exigua*. (A) Effect of dsSeCHY2 on different larval instars by injection or feeding dsRNA. Control dsRNA (‘dsCON’) used EGFP gene. For injection, 3 μg of dsRNA was injected into each larvae. For feeding, 10 μg of dsRNA was fed to each larva. Mortality was determined at 5 days after treatment (ʻ5 DATʼ). (B) Dose-mortality in dsRNA treatment. (C) Time-mortality in dsRNA treatment. Each treatment was replicated three times. For each replication, 10 larvae were used. Different letters above standard deviation bars indicate significant difference among means at Type I error = 0.05 (LSD test).

dsRNA specific to SeCHY2 (dsSeCHY2) was prepared by *in vitro* transcriptional system using T7 RNA polymerase. When dsSeCHY2 was injected, it caused mortalities of late larval instars (Fig 5A). When larvae were fed with dsSeCHY2, they suffered significant mortality, mostly at younger larval stages. The insecticidal activity of dsSeCHY2 was increased with increasing doses in both injection and feeding experiments (Fig 5B). However, the injection method of dsSeCHY2 was much more effective in killing insects than the feeding method. Significant insecticidal activities were observed at 1 μg/larva or more doses in the injection assay. However, 10 μg/larva or more doses were required to achieve the same activities in feeding experiment. Both treatments also interfered with larval development, resulting in reduced body size. The speed to kill was measured after dsSeCHY2 treatment. Significant mortalities were observed at 2 days or later after treatment in both feeding and injection assays (Fig 5C).

**Fig 6:**
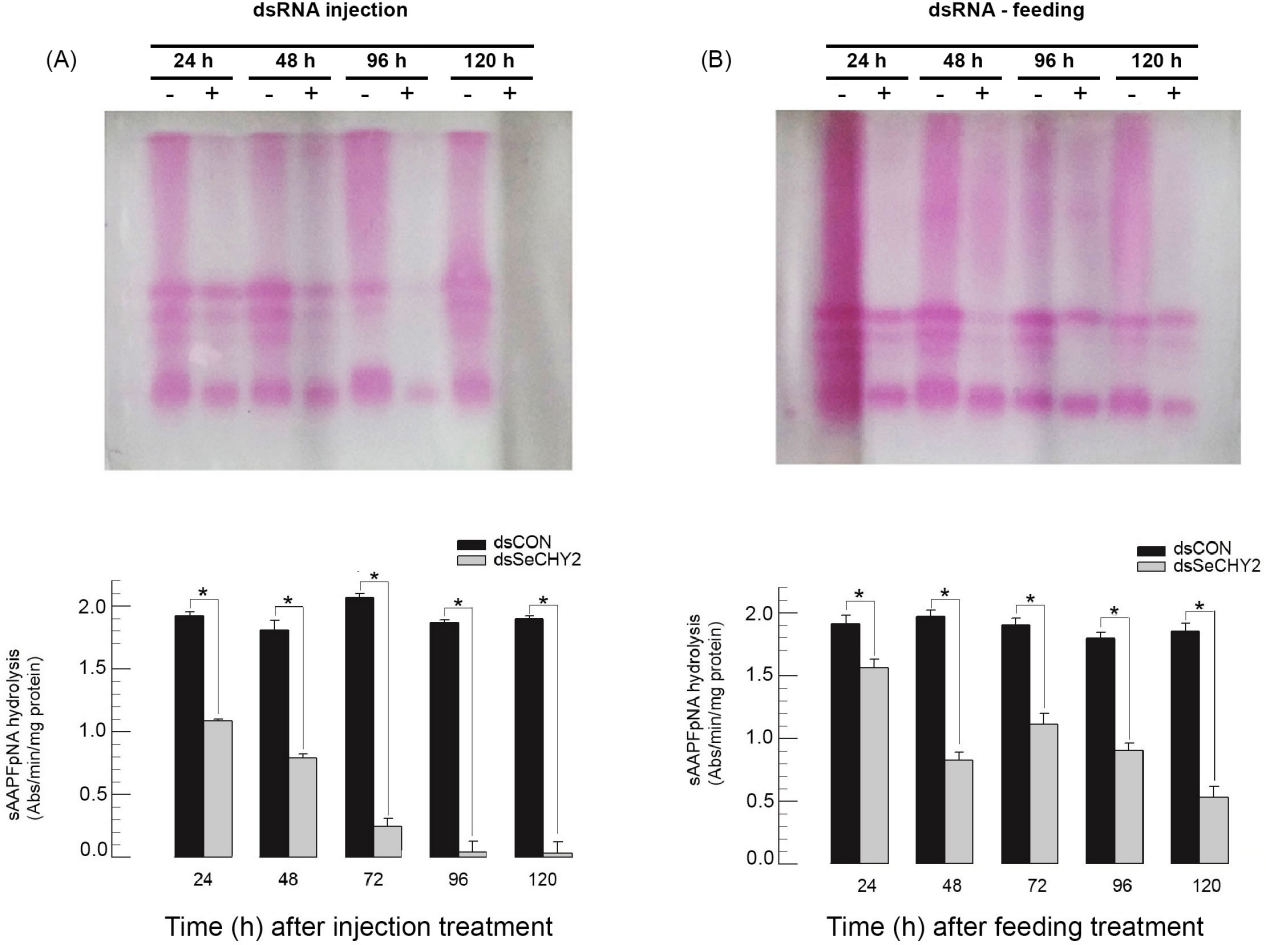
Effect of dsRNA specific to SeCHY2 (‘dsSeCHY2’) on enzyme activity of *S*. *exigua* chymotrypsin in the midgut lumen. (A) dsRNA (6 μg/larvae) was injected to 3^rd^ instar. (B) dsRNA (20 μg/ larvae) was fed to 3^rd^ instar. At different periods, gut lumen was collected and used to visualize (upper panel) or quantify (lower panel) CHY enzyme activity using a fluorescent substrate. Control dsRNA (‘dsCON’) was EGFP gene. For each replication, 10 larvae were used. Asterisk (*) indicates significant difference with control level at Type I error = 0.05 (LSD test).

To understand insecticidal activity of dsSeCHY2, CHY enzyme activity was monitored (Fig 6). In both injection and feeding assays, dsSeCHY2 significantly inhibited CHY activity in the midgut. In injection assays, almost half of chymotrypsin enzyme activity was inhibited at 24 h after dsSeCHY2 treatment and more than 90% of enzyme activity was inhibited after 72 h after injection (Fig 6A). Feeding delivery of dsSeCHY2 was less effective than the injection method. It significantly inhibited CHY enzyme activity at 24 h after feeding. More than 50% of enzyme activity was inhibited at 48 h after feeding treatment (Fig 6B).

### 3.4. dsRNA specific to SeCHY2 possesses potent immunosuppressive activity against *S*. *exigua*

**Fig 7:**
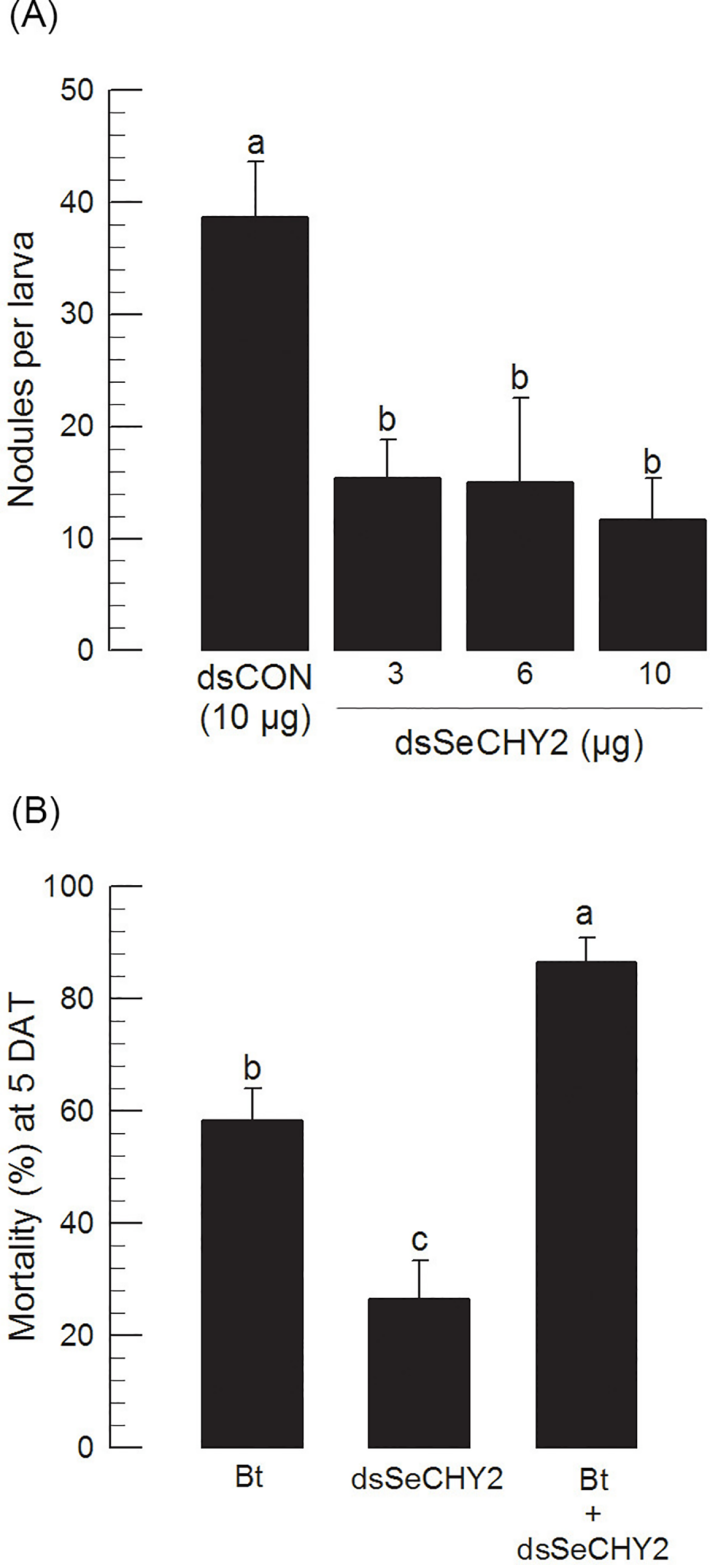
Immunosuppressive activity of dsRNA specific to SeCHY2 against fifth instar larvae of *S*. *exigua*. (A) Nodulation assay. Heat-killed *X*. *hominickii* (5 × 10^5^ cells/larvae) were injected to larva at 48 h after dsSeCHY2 injection. At 8 h post *X*. *hominickii* injection, hemocyte nodules were counted. For each treatment, 5 larvae were used. (B) Enhancement of *B*. *thuringiensis* (Bt) pathogenicity by injection of dsSeCHY2. Bt was treated with 10^5^ ppm by diet-dipping feeding method. Each treatment was replicated three times. For each replication, 10 larvae were used. Different letters above standard deviation bars indicate significant difference among means at Type I error = 0.05 (LSD test).

SeCHY2 was expressed in hemocytes of *S*. *exigua*, suggesting that SeCHY2 might have immunological function. To test this hypothesis, cellular immune response of hemocyte nodulation was assessed after RNAi of SeCHY2 (Fig 7). *S*. *exigua* larvae could form approximately 39 nodules in response to bacterial challenge (Fig 7A). However, nodule formation was significantly reduced in larvae injected with dsSeCHY2 at three different doses. There was little dose-dependency in the reduction of nodule formation. To confirm the function of immunosuppression by dsSeCHY, changes in Bt pathogenicity were monitored (Fig 7B). RNAi with dsSeCHY2 significantly enhanced the insecticidal activity of Bt.

### 3.5. Optimization of oral delivery of dsSeCHY2

**Fig 8:**
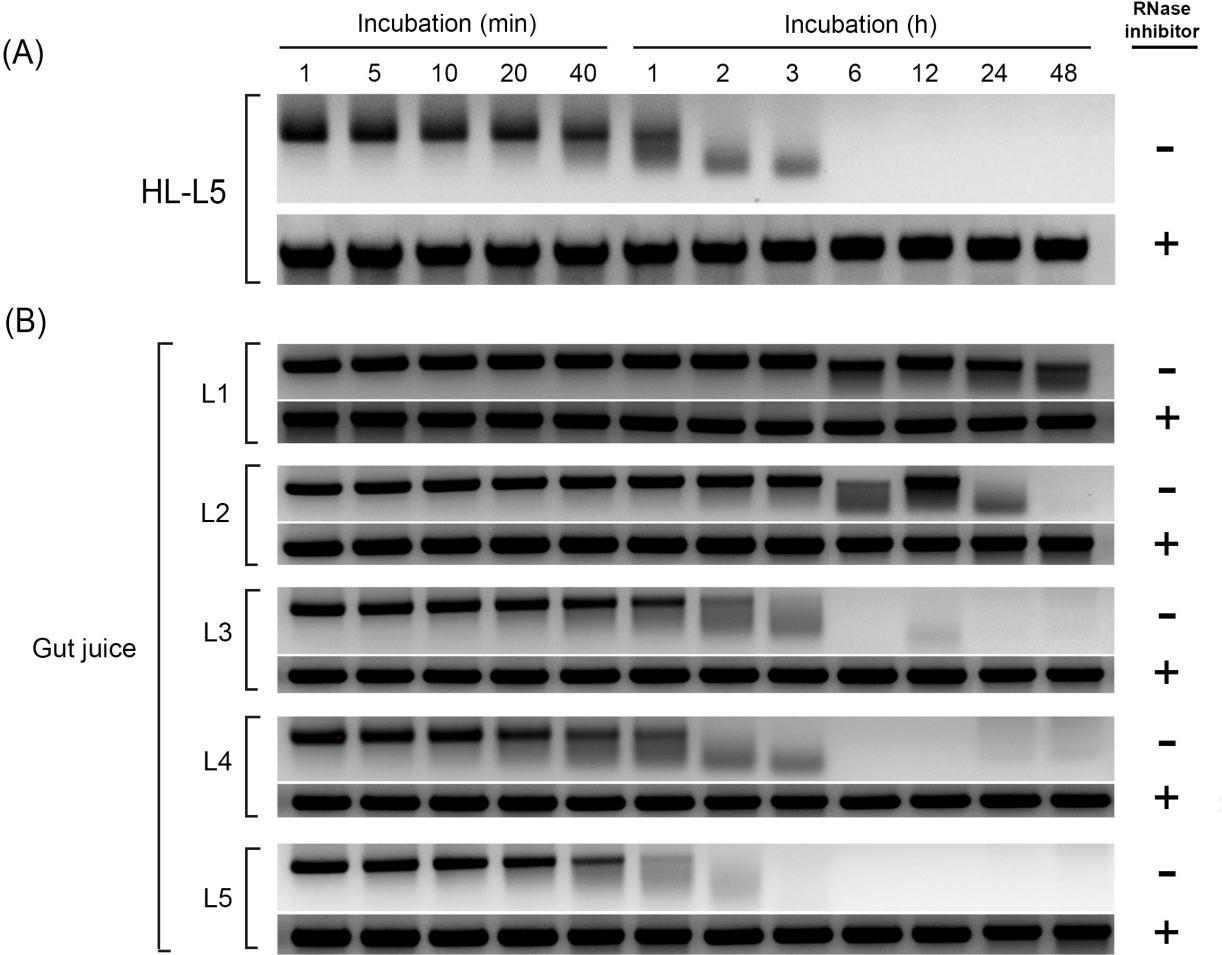
Degradation of dsRNA in hemolymph or midgut juice of *S*. *exigua*. Hemolymph (ʻHLʼ) was collected from fifth instar (ʻL5ʼ). Gut juice was collected from the midgut by collecting supernatant of gut content followed by centrifugation at 10,000 × *g* for 5 min at 4°C. (A) Effect of hemolymph on dsRNA specific to SeCHY2 after incubating 2 μg dsRNA with 10 μl HL sample with different incubation periods. (B) Effect of gut juice on dsRNA specific to SeCHY2 after incubating 2 μg dsRNA with 10 μL gut juice sample. RNase inhibitor (1 μL, 20 ng) was included in the incubation. After incubation, all RNA samples were subjected to 1% agarose gel electrophoresis and visualized with EcoDye DNA staining solution (SolGent, Daejeon, Korea).

It was speculated that the relatively lower insecticidal activity of dsSeCHY2 in feeding delivery compared to that in injection assay might be due to high RNase activity in the gut lumen. Therefore, hemolymph (ʻHLʼ) and midgut digestive juice (ʻGut juiceʼ) were isolated from fifth instar larvae (ʻL5ʼ) and incubated with the same amount of dsSeCHY2. Midgut juice showed high RNase activity. It digested dsSeCHY2 within 1 h of incubation (Fig 8). However, an addition of RNase inhibitor prevented such degradation of dsSeCHY2. Hemolymph sample also exhibited RNase activity. However, it was less active than gut juice. When RNase inhibitor was present, dsRNA samples in HL were protected against degradation. In gut juice, RNase activity appeared to be relatively low in young larval stages. In the first instar larvae (ʻL1ʼ), RNase activity was minimal, in which dsSeCHY2 kept its level without too much change for 2 days.

**Fig 9:**
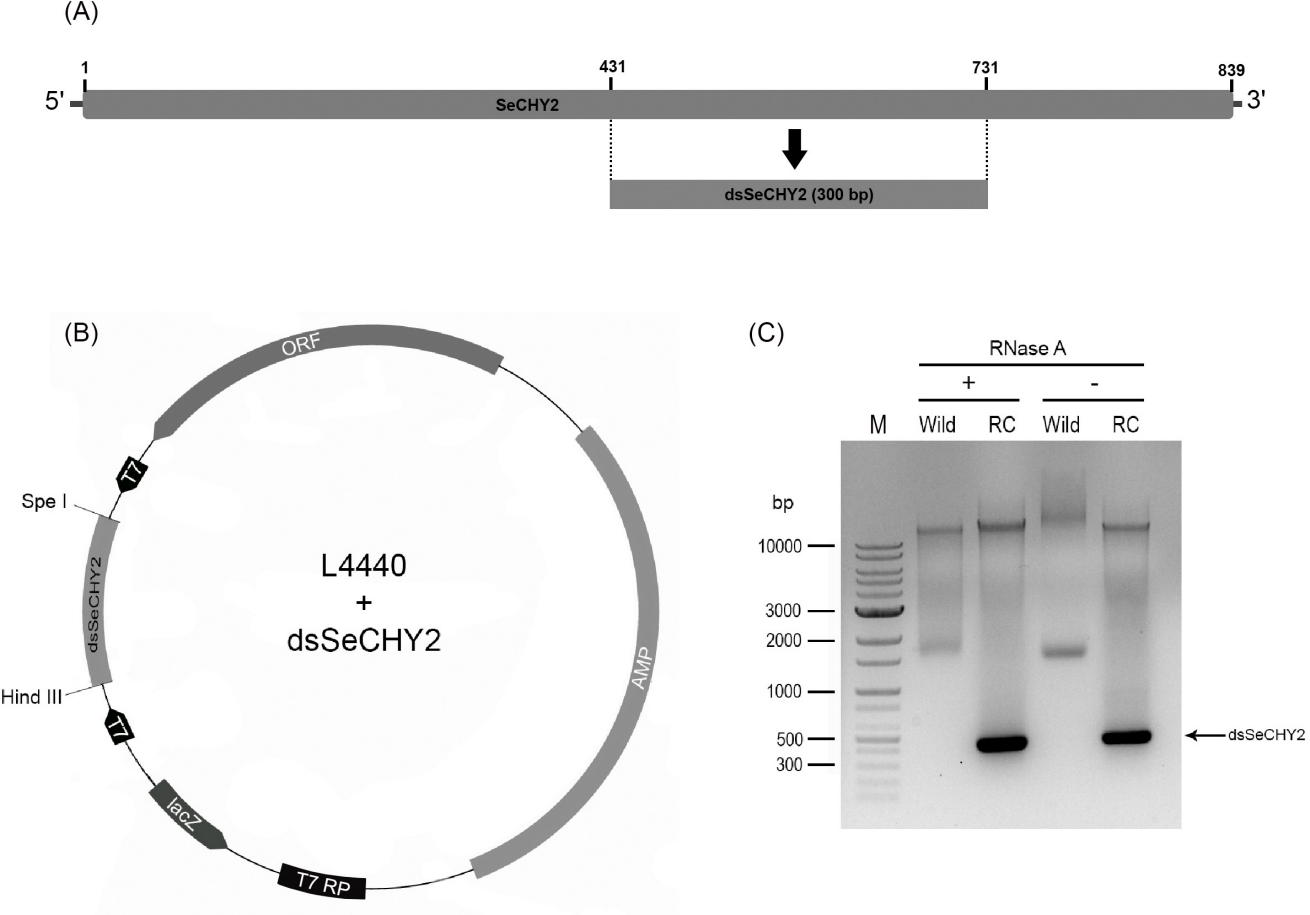
Construction of recombinant *E*. *coli* expressing dsRNA specific to SeCHY2 (dsSeCHY2). (A) Location of dsSeCHY2 in cDNA of SeCHY2. (B) Directional cloning of dsSeCHY2 into L4440 vector using two restriction sites of *Hind*III and *Spe*I. The cloning site was located between two opposite T7 promoters. Recombinant vector was screened after transformation into *E*. *coli* in the presence of ampicillin antibiotics. LacZ promoter (ʻlacZʼ) was then induced by IPTG to express T7 RNA polymerase (ʻT7 RPʼ) which recognized two T7 promoters and transcribed dsSeCHY2 in both directions. (C) Resulting dsSeCHY2 and its resistance to RNase A treatment. ʻWildʼ and ʻRCʼ represent non-recombinant and recombinant HT115 bacteria, respectively. ʻMʼ represents DNA marker.

A bacterial expression system was used to synthesize dsSeCHY2 (Fig 9). dsSeCHY2 was designed at the middle of the gene with size of 300 bp (Fig 9A). This partial gene was inserted into L4440 vector between two T7 promoters by directional cloning using *Hind*III and *Spe*I restriction enzymes (Fig 9B). The recombinant vector was then used to transform bacteria to overexpress dsSeCHY2 through induction by IPTG. RNA extraction from transformed bacteria indicated the presence of dsSeCHY2 which was resistant against RNase A treatment (Fig 9C).

**Fig 10:**
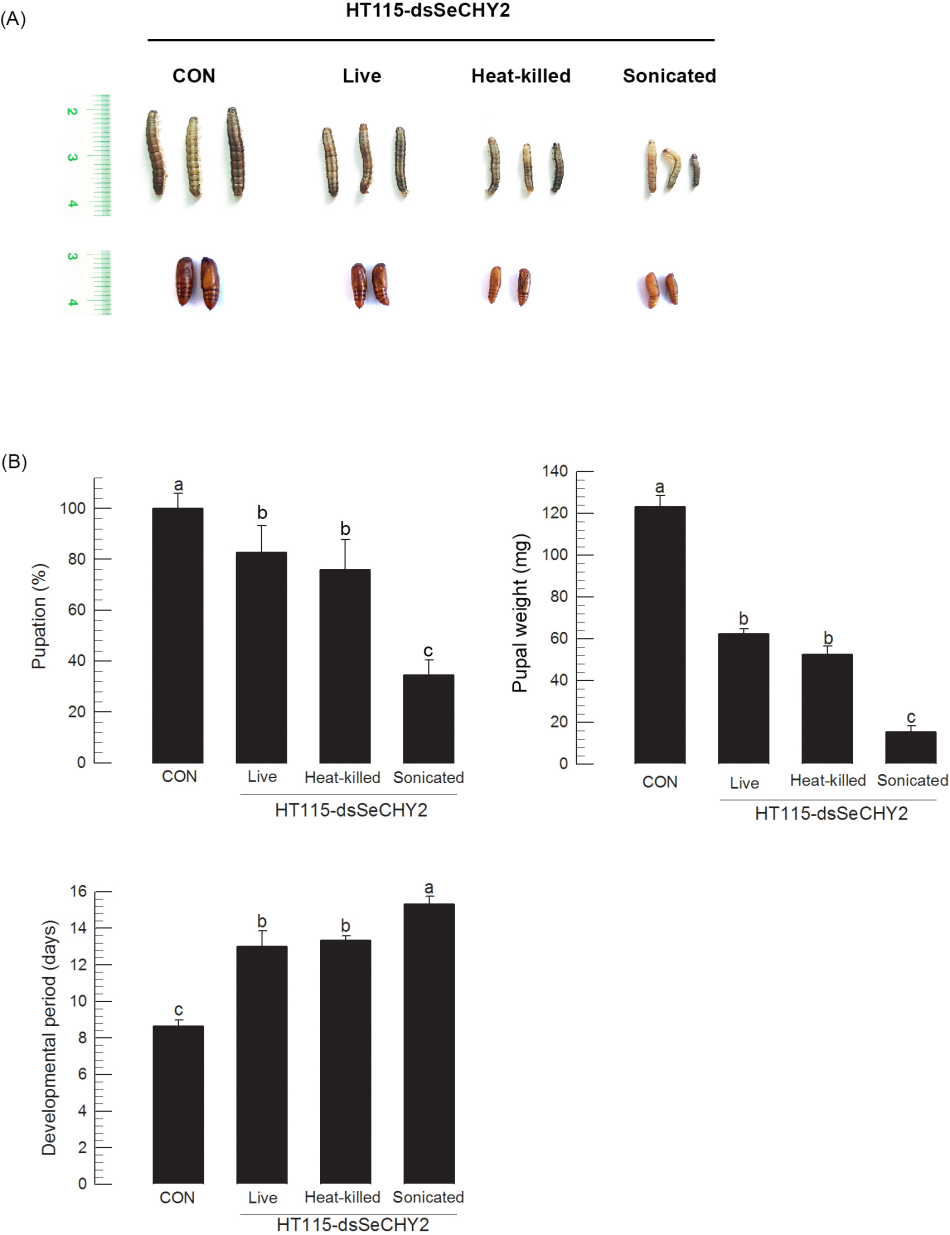
Variation in insecticidal efficacy of *E*. *coli* HT115-expressing dsRNA specific to SeCHY2 (dsSecHY2) after different pretreatments. Pretreatments used no pretreatment (ʻLiveʼ), heat treatment at 95°C for 10 min (ʻheat-killedʼ), or sonication (ʻSonicatedʼ) using ultrasonication to breakdown bacterial cell membrane. Control (ʻCONʼ) used non-recombinant HT115 bacteria for feeding assay. All treatments were used for oral administration of bacteria by loading 10^7^ cells to diet. After feeding all bacteria, larvae were fed with fresh diet for growth. Fourth instar were used as test larvae. (A) Larvae treated with *E*. *coli* HT115 expressing dsSeCHY2 after different pretreatments. (B) Influence of bacterial treatment on different larval developmental parameters. Each treatment was replicated three times. For each replication, 10 larvae were used. Different letters above standard deviation bars indicate significant difference among means at Type I error = 0.05 (LSD test).

To facilitate the release of dsSeCHY2 from transformed *E*. *coli* in insect digestive tract, induced *E*. *coli* cells were pretreated with heat or sonication before oral delivery to *S*. *exigua* (Fig 10). Heat-killed transformed bacteria did not enhance the insecticidal activity compared to live bacteria. However, sonication of bacteria before oral administration significantly increased the insecticidal activity along with adverse effects on larval development (reduced body size and prolonged larval development of treated larvae) (Fig 10B).

**Fig 11:**
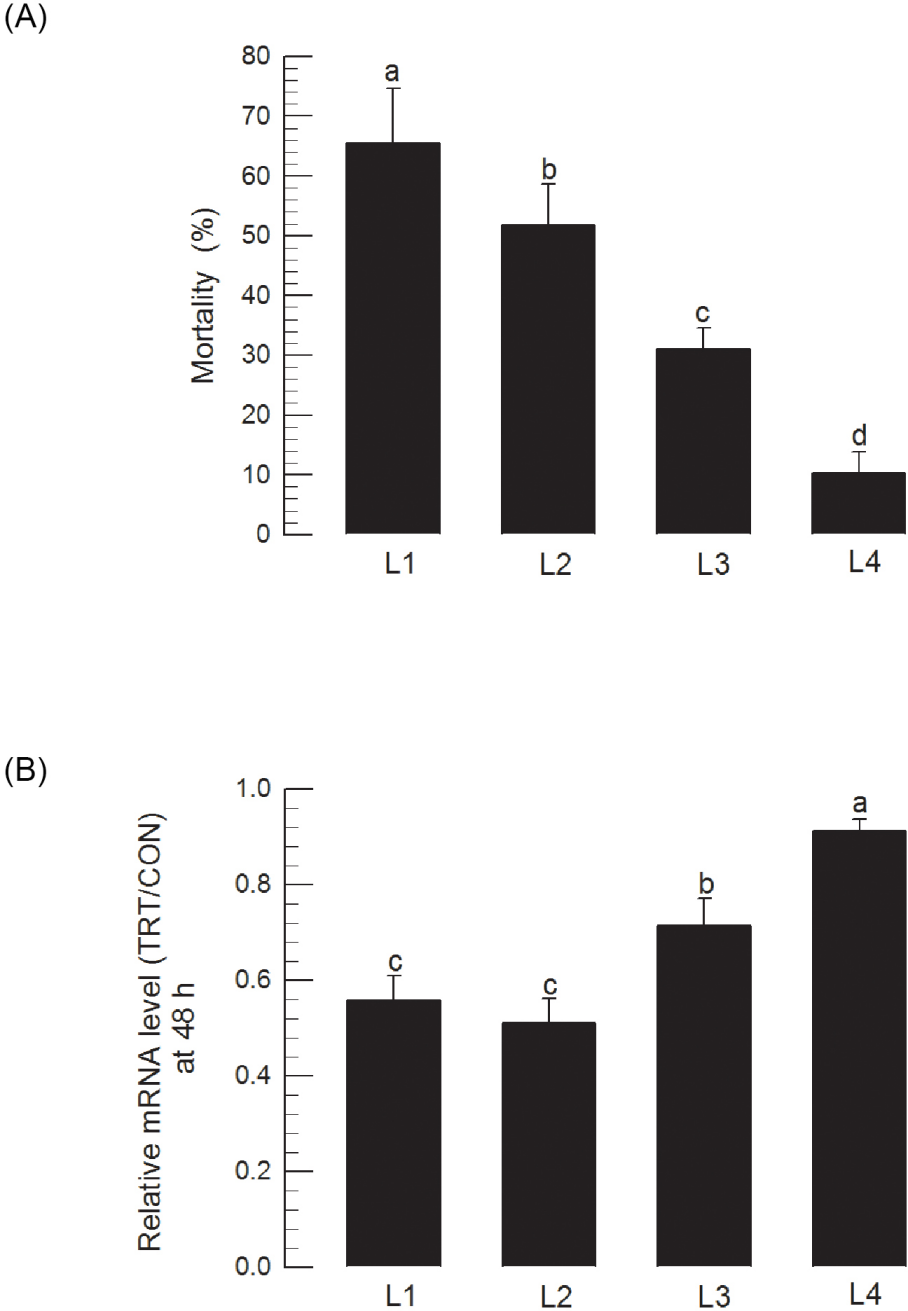
Insecticidal activity of *E*. *coli* HT115 expressing dsRNA specific to SeCHY2 against different larval instars (ʻL1-L4ʼ) of *S*. *exigua*. Sonication-pretreated bacteria were used for oral feeding. Recombinant bacteria were loaded to each diet at a dose of 10^7^ cells. (A) Variation in insecticidal efficiencies of recombinant bacteria depending on developmental stages. Each instar treatment was replicated three times with 10 larvae per replication. (B) Variation in RNAi efficiency of bacteria treatment among developmental stages. In each instar treatment, three individuals were randomly selected and used to measure mRNA levels using RT-qPCR. Each individual was an experimental unit. Relative mRNA was calculated by SeCHY2 level of treatment (ʻTRTʼ) divided by that of control (ʻCONʼ). TRT represents feeding treatment of recombinant bacteria, while CON represents feeding treatment of non-recombinant bacteria. Different letters above standard deviation bars indicate significant difference among means at Type I error = 0.05 (LSD test).

Insecticidal activities of sonicated recombinant *E*. *coli* expressing dsSeCHY2 against different larval instars were assessed (Fig 11). The last instar (ʻL5ʼ) did not show any mortality after oral treatment with dsSeCHY-expressing bacteria. However, insecticidal activity against younger larval instars was increased. At doses of 10^7^ cells or more per larva at L1 stage, 65% of mortality was recorded (Fig 11A). RNAi efficiency was also higher in younger larvae than that in older larvae (Fig 11B).

## 4. Discussion

RNAi technique has been used to analyze gene function by loss of function approach. It also has potential to specifically control target insect pests [25]. However, successful RNAi efficiency has been a challenge, especially for lepidopteran insects [6,26]. There are several breakthroughs to enhance RNAi efficiency and subsequent insecticidal activity in lepidopteran insects [27]. Screening and selecting a promising target gene are highly important to improve insecticidal activity. In this study, CHY was selected as a target gene to construct dsRNA to control *S*. *exigua* because inhibitor assay against different digestive enzymes indicated that CHY-specific inhibitor highly intimidated survival of *S*. *exigua* larvae. CHY is required for larvae growth and development by digesting proteins in the ingested diet [28,29], immune responses [30], and molting procedures [31]. Among seven CHY genes with full ORF encoded and expressed in *S*. *exigua*, SeCHY2 was chosen because it was expressed in all larvae stages and tissues. Furthermore, RNAi efficiency of SeCHY2 was superior to other SeCHYs in both injection and feeding bioassays. Interestingly, RNAi efficiency was clearly observed only in gut at oral administration while it was not in gut at hemocoelic injection. This confirmed that RNAi is not systemic in *S*. *exigua* [6]. This is the first study that reports silence of digestion-associated gene expression in *S*. *exigua*. Midgut-associated genes such as *chitin synthase* [32], *chitinase* [33], and *integrin* [2] have been analyzed by RNAi. They all showed potential as dsRNA-based insecticidal agents. In addition, non-midgut genes of *S*. *exigua* [34] have demonstrated their usefulness for such purpose. SeCHY2 is a digestive enzyme in midgut of *S*. *exigua*. dsSeCHY2 treatment significantly reduced CHY enzyme activity in larval midgut. Oral administration of dsSeCHY2 also resulted in significant mortality of *S*. *exigua* larvae, similar to results obtained after treatment with CHY-specific inhibitor.

To improve RNAi efficiency, efficient dsRNA delivery method is needed. Efficient delivery of dsRNA also needs to stabilize dsRNA in the internal environment (gut lumen or hemocoel) of target insects. dsRNA can be degraded by nucleases in some tissues or gut lumen of insects [6]. Indeed, dsSeCHY2 was degraded in both hemolymph and gut juice mainly by RNase because the addition of RNase inhibitor prevented its degradation. Such dsRNA degradation also occurs in other insects, including lepidopteran species. It is most likely due to specific dsRNA degradation enzyme. For example, a dsRNase in the saliva of *Lygus lineolaris* has been reported to be able to catalyze specific degradation of dsRNA [35]. Pea aphid, *Acyrthosiphon pisum*, also exhibits dsRNA degradation after oral delivery of dsRNA through artificial diet [36]. A specific dsRNase (a member of DNA/RNA non-specific nuclease) in *Bombyx mori* is known to play a crucial role in the digestion of DNA and RNA in the midgut mainly to defend virus attack by degrading viral nucleic acids [37]. In locust (*Schistocerca gregaria*), four different dsRNases have been predicted and RNAi of dsRNase 2 can significantly improve RNAi efficiency of other dsRNAs [38]. These findings suggest that midgut juice of *S*. *exigua* might possess dsRNase activity. Activities of dsRNA degradation varied depending on tissues (hemolymph and gut) and larval development stages of *S*. *exigua*. dsRNA specific to ultraspiracle gene in *H*. *armigera* has shown faster degradation in midgut juice than that in hemolymph [39]. Such differential dsRNA degradation has been explained by more rapid absorption in hemolymph to target cells compared to that in midgut lumen. Therefore, continuous dsRNA ingestion or high amount of dsRNA would be necessary to achieve successful RNAi [39]. Cotton bollworm, *Pectinophora gossypiella*, has shown increased RNAi efficiency with increasing dose of dsRNA [40]. In *Helicoverpa armigera*, when 15 μg of dsRNA specific to transferrin gene is applied with artificial diet, RNAi efficiency is highly varied with elapsed time. However, RNAi efficiency is relatively stable when dsRNA application dose is increased to 35 μg [41].

To stabilize dsSeCHY2 in oral delivery, a transformed *E*. *coli* expressing dsSeCHY2 was constructed initially to deliver dsRNA in this study. Application of recombinant bacterial technology has been performed in *Caenorhabditis elegans* using *E*. *coli* HT115 engineered to knock-out RNase III to improve RNAi efficiency [42,43]. To facilitate the release of dsSeCHY2 from transformed *E*. *coli* in *S*. *exigua* digestive tract, induced *E*. *coli* cells were pretreated with sonication before oral treatment in the present study. Sonication treatment significantly increased RNAi efficiency and insecticidal activity of dsSeCHY2. The sonication treatment gave a physical damage to interfere with bacterial growth probably through impairing bacterial membrane [2]. Thus the damage on the membrane may facilitate the release of dsRNA in the bacteria. In addition, results of this study indicated that the optimal target developmental stages of *S*. *exigua* were young larval instars, for which dsSeCHY2 treatment resulted in 65% mortality. Such activity is higher compared to results of previous dsRNA applications for *S*. *exigua*. For example, recombinant bacteria expressing dsRNA against β-integrin subunit have only resulted in 50% mortality [2]. Targeting digestive genes using dsRNA has been performed in other lepidopteran insects. For example, feeding artificial diet treated with specific dsRNAs to both trypsin and chymotrypsin in *Helicoverpa armigera* has caused only 30% mortality [44]. Thus, a dsRNA delivery system using recombinant bacteria pretreated with sonication can improve the insecticidal activity of dsRNA against lepidopteran insect pests. To further enhance the insecticidal activity, a treatment of bacterial mixture of Bt and *E*. *coli* expressing dsSeCHY2 can be proposed because a similar bacterial mixture increased mortality close to more than 90% [2]. The synergism can be explained by the immunosuppression by dsRNA. This current study demonstrated the immunosuppressive activity of dsSeCHY2.

In summary, a dsRNA was optimized to control a lepidopteran insect, *S*. *exigua*, in this study. To give maximal reduction of survival, a CHY gene was selected. To minimize dsRNA degradation, a bacterial expression and formulation system was utilized. Finally, pretreatment of recombinant bacteria by ultrasonication was found to be able to enhance insecticidal activity by facilitating dsRNA release from these bacteria.

## Supporting information

S1 Table Primer sequences used in this study. (DOCX)

S2 Table GenBank accession numbers and abbreviations used for phylogenetic analysis. (DOCX)

S1 Fig Amino acid alignment of all *S*. *exigua* chymotrypsins (SeCHYs) collected from Spodobase (http://bioweb.ensam.inra.fr/spodobase/). Sequence alignment was performed with Clustal W program and the tree was constructed using MEGA 6.0. Each node contains bootstrap value after 1,000 repetitions. Four subgroups are denoted with A-D.
(TIF)
