# Supplementary material for: Optimization of recombinant bacteria expressing dsRNA to enhance insecticidal activity against a lepidopteran insect, Spodoptera exigua
Source: PLoS One. 2017 Aug 11;12(8):e0183054. doi: 10.1371/journal.pone.0183054 (PMC5553977; doi:10.1371/journal.pone.0183054)
Supplement: S1 Table — (DOCX) [file pone.0183054.s001.docx]

**S1 Table Primer sequences used in this study**

| Primer | Uses | Orientation | Sequence (5ʹ - 3ʹ) | Annealing temperature (ºC) | Size (bp) |
| --- | --- | --- | --- | --- | --- |
| SeCHY2 | RT-qPCR | Forward | GTCCTCATCACCTCCTCAAAC | 50 | 300 |
|  |  | Reverse | CAGAGTCACCGTTGCAAGTA |  |  |
| SeCHY3 | RT-qPCR | Forward | CAAGAGTCCTGGTTCTCAACTC | 50 | 525 |
|  |  | Reverse | TCTGGTGTTCACCACCTTTC |  |  |
| SeCHY4 | RT-qPCR | Forward | GCAGTCGTTGCCTTCAATAGTA | 50 | 389 |
|  |  | Reverse | TCTGTGGAGGTTCTCTGATCTC |  |  |
| SeCHY6 | RT-qPCR | Forward | GAACAGCGGTACGGAATACA | 52 | 523 |
|  |  | Reverse | CTAGACGATGACAGACCTCAAC |  |  |
| SeCHY10 | RT-qPCR | Forward | TTCTGTGGTGGTTCCTTGATAG | 52 | 476 |
|  |  | Reverse | TGTAAGGTGCAGACCTCAATAC |  |  |
| SeCHY18 | RT-qPCR | Forward | CCTTACCTTCTGCTGTGTCTAC | 52 | 328 |
|  |  | Reverse | GAGGTCACGCCAATCAAGATA |  |  |
| SeCHY19 | RT-qPCR | Forward | GCCACTAGCAGACAACATGATA | 52 | 373 |
|  |  | Reverse | CTGGGAATCCTACAGCACAAG |  |  |
| dsSeCHY2 | dsRNAi | Forward | TAATACGACTCACTATAGGGAGAGGTCCTCATCACCTCCTCAAAC | 50 | 325 |
|  |  | Reverse | TAATACGACTCACTATAGGGAGAGCAGAGTCACCGTTGCAAGTA |  |  |
| dsSeCHY3 | dsRNAi | Forward | TAATACGACTCACTATAGGGAGACAACCTGTGCCTCTCACTTAT | 50 | 336 |
|  |  | Reverse | TAATACGACTCACTATAGGGAGAGAGACGATACCGAACTGATGAC |  |  |
| dsSeCHY4 | dsRNAi | Forward | TAATACGACTCACTATAGGGAGACCAGCAACTCTAGCTTGTACTC | 50 | 298 |
|  |  | Reverse | TAATACGACTCACTATAGGGAGATCTCACAGCAGCTCATTGTATC |  |  |
| dsSeCHY6 | dsRNAi | Forward | TAATACGACTCACTATAGGGAGAGACCCATCCTGACTACGATTT | 50 | 299 |
|  |  | Reverse | TAATACGACTCACTATAGGGAGAGTACGCCATGTTCAGTGTTATG |  |  |
| dsSeCHY10 | dsRNAi | Forward | TAATACGACTCACTATAGGGAGAGTCGTATTGTCGGTGGTACTG | 50 | 303 |
|  |  | Reverse | TAATACGACTCACTATAGGGAGACATGAAGTCGTAGTCTGGATGG |  |  |
| dsSeCHY18 | dsRNAi | Forward | TAATACGACTCACTATAGGGAGAAGCCCGACTTGGTCAATTT | 50 | 316 |
|  |  | Reverse | TAATACGACTCACTATAGGGAGAGTAGACACAGCAGAAGGTAAGG |  |  |
| dsSeCHY19 | dsRNAi | Forward | TAATACGACTCACTATAGGGAGTTCTTCGAGTCGGGTTATGT | 50 | 315 |
|  |  | Reverse | TAATACGACTCACTATAGGGAGCAATAGCCTTCGTGAATGTG |  |  |
| RB-dsSeCHY2 | Recombinant bacteria | Forward | CCCACTAGTGTCCTCATCACCTCCTCAAAC | 50 | 318 |
|  |  | Reverse | CCCAAGCTTCAGAGTCACCGTTGCAAGTA |  |  |
| dsCON | Control dsRNA | Forward | CCCACTAGTGTCCTCATCACCTCCTCAAAC | 50 | 520 |
|  |  | Reverse | CCCAAGCTTCAGAGTCACCGTTGCAAGTA |  |  |
| dsCON | RT-PCR, qPCR | Forward | CCCACTAGTGTCCTCATCACCTCCTCAAAC | 50, 52 | 273 |
|  |  | Reverse | CCCAAGCTTCAGAGTCACCGTTGCAAGTA |  |  |
