# Supplementary material for: Optimization of recombinant bacteria expressing dsRNA to enhance insecticidal activity against a lepidopteran insect, Spodoptera exigua
Source: PLoS One. 2017 Aug 11;12(8):e0183054. doi: 10.1371/journal.pone.0183054 (PMC5553977; doi:10.1371/journal.pone.0183054)
Supplement: S2 Table — (DOCX) [file pone.0183054.s002.docx]

**S2 Table** **GenBank accession numbers and abbreviations used for phylogenetic analysis**

| Species | Abbreviate | Gene Name | GenBank accession number | |
| --- | --- | --- | --- | --- |
| *Anopheles gambiae* | Ag | chymotrypsin | Z18887 | |
| *Aedes aegypti* | Aa CTP II | chymotrypsin II-like protein precursor | AF237415 | |
|  | Aa | chymotrypsin-like serine protease (JA15) | AY957559 | |
|  | Aa L | larval chymotrypsin-like precursor | AF487334 | |
| *Culex pipiens pallens* | CpP | putative chymotrypsin-like protein | AF468495 | |
| *Glossina morsitans* | Gm | morsitans chymotrypsin-like protein (chy1) | EU589385 | |
| *Phlebotomus papatasi* | Pp chym1 | chymotrypsin (chym1) | AY128106 | |
| *Lutzomyia longipalpis* | Ll Chym 1A | putative chymotrypsin (Chym1A) | EU124576 | |
|  | Ll Chym 1B | putative chymotrypsin (Chym1B) | EU124575 | |
|  | Ll Chym 2 | putative chymotrypsin (Chym2) | EU124583 | |
|  | Ll Chym 3 | putative chymotrypsin (Chym3) | EU124591 | |
|  | Ll Chym 4 | putative chymotrypsin (Chym4) | EU124573 | |
|  | Ll Chym 5 | putative chymotrypsin (Chym5) | EU124574 | |
| *Phlebotomus papatasi* | Pp Chym 2 | chymotrypsin (chym2) | AY128107 | |
| *Bombyx mori* | Bm P | chymotrypsin-like proteinase | JQ081296 | |
|  | Bm | chymotrypsin-like serine protease | NM_001046965 | |
| *Spodoptera frugiperda* | Sf | chymotrypsin precursor | AY251276 | |
| *Spodoptera litura* | Sl CTLP2 | chymotrypsin-like protein 2 (CTLP2) | GQ891130 | |
|  | Sl | chymotrypsin-like protein precursor | GQ354838 | |
| *Helicoverpa armigera* | Ha | clone HaFLS01825 chymotrypsin | EU325550 | |
| *Amyelois transitella* | At | chymotrypsin BII-like | XP_013183860 | |
| *Ostrinia furnacalis* | On CTP16 | Midgut chymotrypsin | AFM77775.1 | |
| *Diatraea saccharalis* | Ds | chymotrypsin 2b | AFW03966 | |
| *Danaus plexippus* | Dp | chymotrypsin | EHJ67193 | |
| *Stomoxys calcitrans* | Sc | chymotrypsin | AY190632 | |
| *Papilio xuthus* | Px | chymotrypsin | KPJ01456 | |
| *Papilio machaon* | Pm | chymotrypsin | KPJ18047 | |
| *Helicoverpa zea* | Hz | HzC4 chymotrypsinogen | AAF71518 | |
| *Helicoverpa punctigera* | Hp | chymotrypsinogen | AAV33655 | |
| *Heliothis virescens* | Hv | chymotrypsin | AFM28261 | |
| *Mythimna separata* | Ms | chymotrypsin | AKR06192.1 | |
| *Agrotis ipsilon* | Ai | AiC5 chymotrypsinogen | AAF71516 | |
| *Ostrinia nubilalis* | On CHY1 | chymotrypsin-like serine protease 16 | AFM77775 | |
| *Papilio xuthus* | Px CHY2 | Chymotrypsin-2 | KPI90986 | |
| *Bombyx mori* | Bm CLP | chymotrypsin-like proteinase | AFD99127 | |
| *Manduca sexta* | Ms CLP3 | chymotrypsinogen-like protein 3 | CAM84318 |  |
