# Supplementary figures and images for: Optimization of recombinant bacteria expressing dsRNA to enhance insecticidal activity against a lepidopteran insect, Spodoptera exigua
Source: PLoS One. 2017 Aug 11;12(8):e0183054. doi: 10.1371/journal.pone.0183054 (PMC5553977; doi:10.1371/journal.pone.0183054)

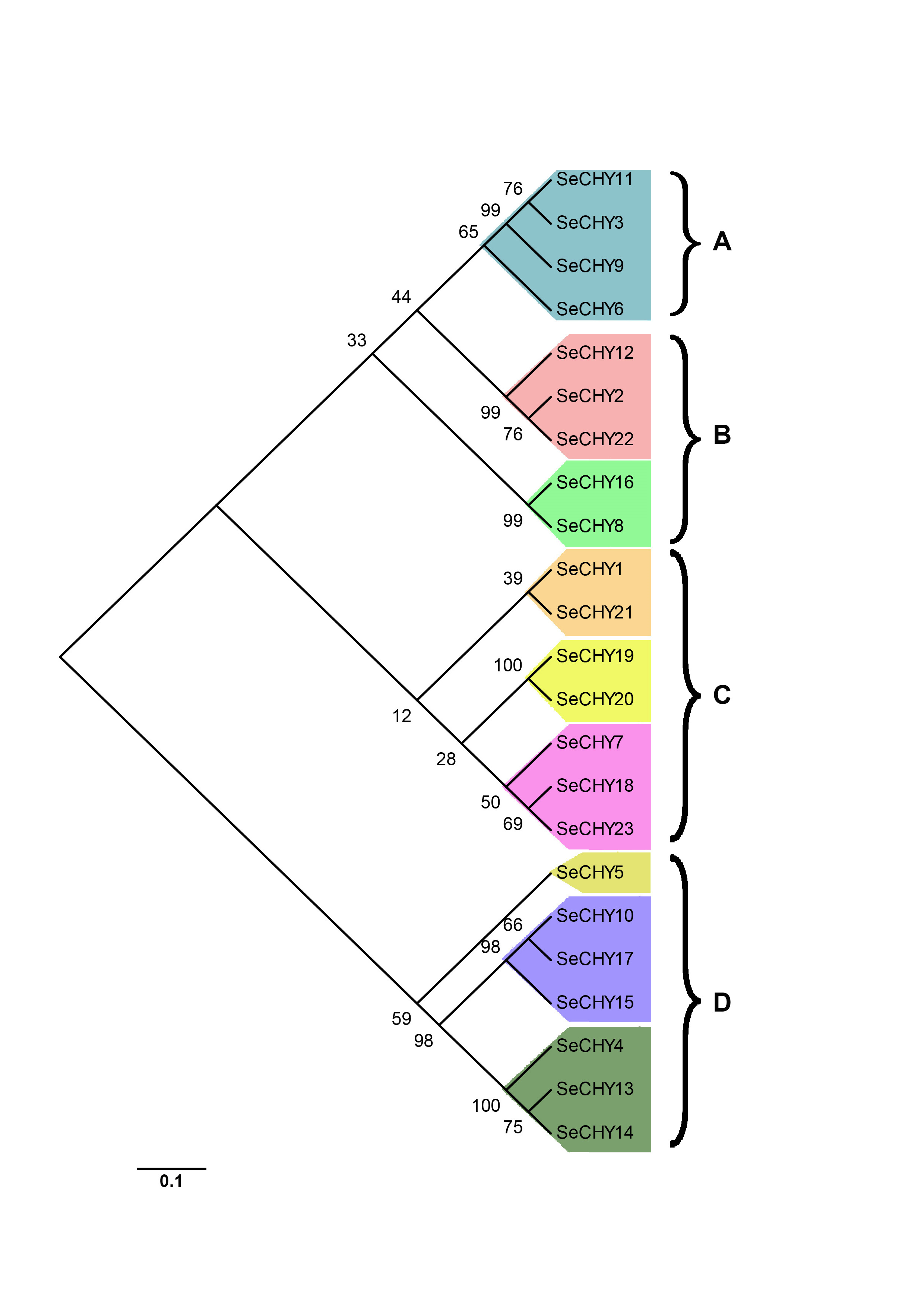

Supplement: S1 Fig — Sequence alignment was performed with Clustal W program and the tree was constructed using MEGA 6.0. Each node contains bootstrap value after 1,000 repetitions. Four subgroups are denoted with A-D. (TIF) [file pone.0183054.s003.tif]
